# A multi-dimensional student performance prediction model (MSPP): An advanced framework for accurate academic classification and analysis

**DOI:** 10.1016/j.mex.2024.103148

**Published:** 2024-12-30

**Authors:** V. Balachandar, K. Venkatesh

**Affiliations:** Department of Networking & Communications, School of Computing, SRM Institute of Science and Technology, Kattankulathur, Chennai, India

**Keywords:** Classification, Deep learning, Personalized learning, Feature engineering, Explainable AI, Artificial neural network, Multi-dimensional Student Performance Prediction Model (MSPP)

## Abstract

Forecasting student performance with precision in the educational space is paramount for creating tailor-made interventions capable to boost learning effectiveness. It means most of the traditional student performance prediction models have difficulty in dealing with multi-dimensional academic data, can cause sub-optimal classification and generate a simple generalized insight. To address these challenges of the existing system, in this research we propose a new model Multi-dimensional Student Performance Prediction Model (MSPP) that is inspired by advanced data preprocessing and feature engineering techniques using deep learning. We developed a method that targets the common issues associated with educational datasets over imbalanced and temporal settings which is also explainable through AI features. Moreover, through adaptive hyper-parameter tuning and advanced graph neural network layers in the MSPP model allow to make output more dense representation for predictions resulting more accurate classification. The experiments results show that MSPP outperforms the other EAI&ML, MTSDA, XAI, DGNN and DLM with high accuracy 76 %, precision score of 0.79 and macro F1-score of 0.73. The model also helps to bring down the False Positive Rate (FPR) substantially at a 0.15 level, which ensures more reliable predictions for student classification.

• The model of the MSPP includes contextual information and multi-layered analysis in order to improve prediction accuracy, placing a sound basis for predicting students in different performance categories such as distinction, pass, fail or withdrawn.
• Our approach is obviously to generalize and extract those sparse, heterogeneous academic data in the form of structured training record using domain specific preprocessing integrating with multi-class classification mechanisms that improves on precision-recall across multiple categories.

## Graphical abstract

**Figure:**
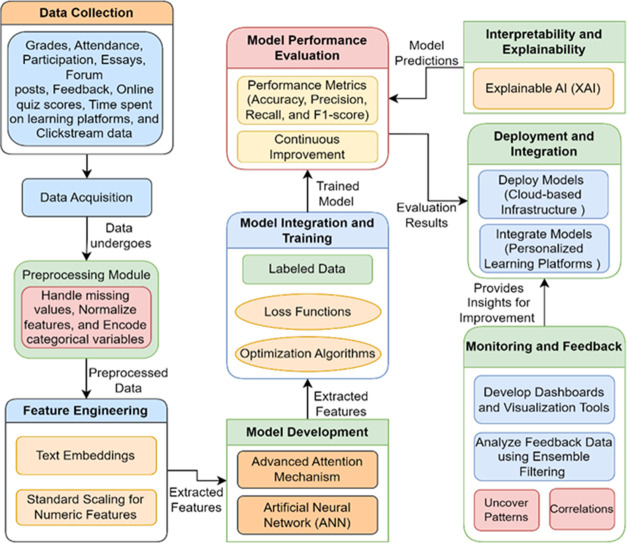

Specifications table

**Table:** 

Subject area:	Engineering
More specific subject area:	Conventional predictive models - Forecasting student performance
Name of your method:	Multi-dimensional Student Performance Prediction Model (MSPP)
Name and reference of original method:	Personalized Learning, Feature Engineering, Explainable AI, Artificial Neural Network
Resource availability:	*N.A.*

## Background

### Introduction

In the rapidly evolving field of education, data analytics is proving to be a game changer by providing innovative solutions for predicting student outcomes and delivering customized learning [1]. Educational institutions have growing recognized the necessity of data-driven insight in identifying pattern, trends, and correlations that can help educators to take actionable decisions [2]. Yet traditional methods of predicting student performance often involve standardized test scores or established metrics of assessment, which — while informative for certain aspects— fall directly into predefined categories. Most of these traditional techniques are based on superficial indicators such as grades and test scores which do not account for the plethora of factors that contribute to a students' learning journey, including socio-economic background, cognitive diversity in particular areas (i.e. logic/ linguistics), variances in how they prefer to learn material best perceived yet failed by faculty/staff or external influences [[3], [4], [5]]. Consequently, these methods tend to result in broad insights lacking the specificity and complexity of specific student performance.

A major drawback of conventional predictive models is their struggle to capture the inherent versatility within student populations. Modern classrooms are diverse based on learning preferences, cultural backgrounds, prior knowledge and socio-emotional requirements. These differences have been hard for conventional models, which tend towards one-size-fits-all predictions outpatient doctors battle to match. This leads to result in performance prediction of human which is not according on their own unique learning context and Learning Needs. For example, two students who score similarly on a test may have very different paths of learning as controlled by motivation, support systems or other forms showing cognitive engagement [6]. These subtle nuances that traditional models are unequipped to sort, may result in either generic interventions or those not aligned to the actual need of students. As a result, they miss the dangerous chance to intervene specifically which happen very likely will make more difference in student educational outcomes for at-risk youth than just about anything else. Additionally, the cons of conventional strategies pour over to when and how accurate performance predictions happen. Recognising who is most a risk of academic underperformance as early and exactly as possible is critical to enable the timely intervention needed, yet traditional models on their own often prove inadequate. Such methods tend to analyse static data points extracted at predetermined intervals (e.g. when an exam is given), but they are not enough as by nature, student learning is a dynamic and embryonic process Consequently, the models built using this subset of demographic data make predictions based on a reactive rather than proactive time frame resulting in delayed notifications that weaken the efficacy of educational support strategies. For instance, by the time an at-risk student is picked up for low GPA, understanding him/herself as doing poorly in core academic work and thus the interventions are less likely to work. The time lag of the predictions is a substantial missed opportunity to intervene earlier, before those challenges grow larger and undermine the goal as a whole—to improve student success.

The purpose of the work is to construct a powerful predictive student performance model using state-of-the-art data workflows. In this study, a new model has been introduced called the Multi-dimensional Student Performance Prediction Model (MSPP) to break free from these limitations by using multi-layer feature extraction followed by expert's domain specific data pre-processing and incorporating context-based information. Its ten main goals are academic achievement, predicting student outcomes, personalized learning experiences, identifying at risk students and promote equity and inclusion. In addition to these goals, the model also seeks to protect data privacy and security of information collected on individual children; improve decision making by holding stakeholders accountable for meeting educational objectives in which they can influence or control outcomes; foster continuous improvement at every level; enable educational research within a legal framework that protects student confidentiality but still allows examination of hard-to-scrutinize questions about what works best for different kids under varying conditions. Meeting these goals further sets up MSPP to provide not just insights but likewise enables educational institutions to make more prudent and in line with the unique requirements as well as challenges of their student populations. The MSPP model we propose, has several unique contributions due to its ability of incorporating domain-specific knowledge and multi-dimensional analysis making the predictions more specific and coherent. Unlike the usual models, MSPP considers a large area of factors that impact student outcomes – not only demographics and previous academic statistical data, but also behavioural information and other contextual dimensions. The model achieves state-of-the-art performance across a variety of evaluation metrics using its distinctive feature engineering strategies along with its ability to generalize more flexibly over diverse datasets. MSPP also incorporates explainable AI methods, providing insights into the black-box output for educators and administrators. The remainder of this paper is organized as follows: Section 2 reviews related work and existing methodologies in student performance prediction. In section 3, we describe the MSPP model proposed and its architecture, feature extraction processes as well as multi-dimensional examination techniques. In Section 4, we present the results and perform a comparison of MSPP vs traditional models based on multiple performance metrics. Section 5 closes the paper with insights on prospective research and applications of MSPP model.

### Related works

Several practices have been studied in the student performance prediction domain including graph-based neural networks, traditional machine learning models, time series analysis and ensemble learning. Any other traditional way cannot be used for this complex student data otherwise: However, with limited accuracy, adaptability and personalization in contrast to the diversity of it there are many challenges faced by any other approach. This review highlights key contributions from recent literature while identifying their limitations and contrasts them with the enhancements introduced by our proposed model. Huang et al. [7] utilize dual graph neural networks to integrate both interaction activities as well as the attribute features of students. So, they have to hybrid what local online interaction-based academic achievements with global representations from individual students. Especially, it constructs multi-dimensional data and dynamically perform graph convolution operation more effectively than GRL operator, at least for now. The proposed method may be suitable to capture rich-featured properties in the low rank space rather than some conventional powerful operators while those operations are still computationally expensive or less-scalable on large scale applications due to fixed sizes of structure preserving models like a GCN or an LGCN model etc. Instead, its representation learning might fail to understand the temporal relationships over different periods of intensive care. Our work improves on this by utilizing a multi-layer approach accommodating both computational efficiency and robust feature extraction.

Guleria et al. [8] proposed a framework for career counselling through ML and AI targeting job placements and skilling. The White Box and the Black Box models bring interpretability, especially in high-stakes environments. In contrast, the scope of their work references is quite general and concentrates mainly on employability, which may not be easily adaptable to predicting overall academic performance. Our solution extends to a broader range of academic metrics while maintaining the model explainability. Fazil et al. [9] A student performance prediction system based on a convolutional BiLSTM network. The model is built to effectively handle a batch of multiple sequential behavior vectors and applies attention mechanisms for selecting the relevant features. Given the complexity of expertise, ASIST is definitely at its best where we have a lot of behavioral data (clickstreams or midterms) and many opportunities to observe both experts and novices in action. Moreover, deep learning models similar to ASIST can be inefficient in terms of explainability. Our model was designed to maintain a balance between the complexity of features and transparency, allowing better generalization across diverse educational contexts. Shou et al. Wang et al. [10] also design a multi-dimensional time-series model about student learning records, exams and demographic features of students. The model's strength lies in capturing temporal correlations and multidimensional characteristics to predict performance. However, time-series models often face challenges with scalability and can be prone to overfitting in high-dimensional settings. Our work extends this by integrating hybrid time-series analysis with feature selection techniques, improving both accuracy and model robustness. Jang et al. [11] leverage explainable AI (XAI) techniques alongside machine learning models to predict academic performance. They incorporate qualitative insights from educational stakeholders, which enhances feature selection and model relevance. However, the reliance on qualitative data and XAI approaches may limit scalability and model precision in larger or more diverse datasets. Our model improves interpretability while maintaining high predictive accuracy by combining XAI with advanced feature engineering.

Karthikeyan et al. [12] introduce a hybrid educational data mining (HEDM) model aimed at enhancing educational quality through performance analysis. While effective, the model's hybrid nature introduces complexity that may not be necessary for certain applications, leading to potential overfitting. At the other end of this spectrum, our proposal simplifies the downstream workflow by adopting few globally inter-connected clustering methods with both high accuracy in minimizing model complexity. Ramaswami et al. [13] examines difference classifiers using process mining attributes to forecast student outcomes. Process mining improves predictive accuracy, but at a cost in settings that require real-time predictions. Because the contexts within which we are working change, we designed our algorithm to be both speedy and accurate enough that it is useful in real-world educational settings. Adekitan et al. [14] applied certain data mining algorithms to study the effect of admission criteria on first-year academic performance. Such models might not possess the required level of flexibility over time and their ability may lessen or vanish in predicting the long-term performance, but often suitable for identifying initial academic trends. Unlike any of the already established predictive learning analytics approaches, our approach goes beyond being a data repository and investigating static models that were built using initial student information. Ho et al. [15] is the first to study predictors of undergraduate student satisfaction by means of machine learning and regression analysis. It means their approach with recursive feature elimination methods has helped the model performance, but may not have captured deeper behavioral patterns influencing academic success. Our model fills this void by integrating behavioral analytics with the feature selection phase. Ramaswami et al. [16] proposed a generic predictive model with using the CatBoost algorithm. Although it does perform well, particularly on diverse educational datasets composed of many multiple-choice questions, single algorithm usage may potentially be restricting the adaptability to distinct scenarios. This is the first work that proposes a modular framework with algorithmic flexibility, which can be customized based on dataset characteristics. Hasib et al. [17] focused regarding to the secondary education success forecasting in technique study. While this method works well for binary and multi-class classification problems, it can fall short in managing with imbalance datasets as well as complicated real-world situations. This we tackle by advanced sampling techniques and ensemble models which generally perform better when the data is imbalanced.

Raj et al. [18] predicts early-phase student engagement via virtual learning environment data. It points out excellent use-cases for retrospective evaluation but the drawback is how slow they are, especially when you think that in a moving target index like trying to optimize CVR from month-to-month. To maintain correctness in fast paced learning environments, our model accommodates real-time data streams. Asselman et al. [19] introduces ensemble learning models for incorporating post data analysis with known predictions returning. Although the ensemble methods have proven to be more successful, they come with a price of bringing redundancy and adding computational overhead. We design ensemble methods to improve upon this by combining only appropriate models together, maintaining the predictive gain while eliminating any overlap. Mengash et al. [20] explore data mining technics in university admissions, by predicting academic performance of students. As a model built for admissions, it is less flexible to academic contexts outside of its specified use case. So, the full text would read: With all components of our work extending outside admissions, providing a universal method for predicting performance over the student lifecycle. Bujang et al. [21] Machine-learning models to predict final-semester grades; challenges of imbalanced multi-classification. While SMOTE (Synthetic Minority Oversampling) is used so that there are enough positive examples for the adversarial loss, overfitting is a concern. We improve these two parts by balanced sampling and advanced regularization techniques to prevent overfitting, also maintain model accuracy. Ouatik et al. Big Data [22] is put to be good use for faster processing of educational data with ensuring execution time. However, this can decrease the processing time but may reduce quality of prediction if we must predict in a near real-time. This middle of the road solution is both fast yet not too liberal in its distribution to compromise accuracy. Literature review highlights substantial progress but also limitation in scalability, adaptability and generalizability. Work in our manuscript addresses these gaps presenting a novel, hybrid and scalable model that combines state-of-the-art feature selection methods with adaptive clustering techniques to deliver an accurate personalised prediction of student performance for better targeting timely -based interventions.

## Method details

In this work, we focus our effort on such a proposal using state-of-the-art techniques in machine learning to predict student performance. During the first step of our work, we start by collecting different data sources — such as performance scores (e.g., grades), attendance records, participation metrics or any other source provided publicly through students' responses in essays or forum posts; feedback received which can be from professors and peers on assignments given; online quiz results if available beyond what's graded offline; time spent learning platforms per student when they're synchronous activities not attributable to engage learners within an appropriate timeframe due either some commitments outside class hours where internet connectivity could automatically track engagement without being actively required. Preprocess this wide dataset to handle missing values, convert categorical and normalize the features such that it is analysis ready. Then we go to feature engineering where suitable features are extracted from the data that can improve prediction accuracy. This encompasses text embeddings from student essays and forum posts, social network structures, temporal features as well as real-time parameters like quiz scores online/learning platform time spent. In our case, we use text embeddings such as Word2Vec, GloVe and FastText, while numeric features are scaled using traditional scaling.

### Motivation

Given the impetus for using data analytics to predict student performance, certain important questions need answers as they tend to blossom with innovation and improvement. The primary goal is, of course, to improve education quality using data analytics to drive academic success and learning overall. In these data-rich educational environments today, the power of probing student performance through shutdown enables educators and policymakers to make decisions based on strength sitting from state challenges striving for more effective intervention. This insight aids student achievement by providing evidence-based strategies specific to the unique profiles of struggling learners. Customizing learning experiences constitutes one of the biggest advantages of educational data analysis. Through the examination of individual student data, instructors can tailor instruction and resources to encounter each student's inimitable learning preferences, strengths, as well as the growth of their areas. Personalized learning has been shown to increase involvement, motivation, and ultimately, student success by delivering personalized educational pathways that resonate with learners on a more personal level [23]. Furthermore, data analytics is crucial for the early detection of vulnerable pupils, enabling instructors to take specific action before academic difficulties become unmanageable. So having timely and targeted predictive analytics that will provide interventions way ahead the time when it is already showing signs of decline academically can address more in preventing academic drop, but improve to continue growing. In addition, it is also a positive motivation for resources optimization inside education institutions. Taking into account details of how students are performing will help schools allocate resources that keep nutrition services and intervention where they need to be. This targeted funding not only advances academic standards but, also ensures no children are left out of the system because they lack the resources or attention [24]. The ability of data to foster innovation is equally compelling — it can illuminate new trends and better pedagogy while pinpointing areas in need of works. Striving towards continuous improvement, these data-driven feedback loops drive ongoing enhancements in educational quality and effectiveness.

Democratizing data is a similar spur for EdTech, giving all students access to the same support and beneficial resources. Examining student performance data can help identify differences in educational outcomes, and appropriate actions must be taken to ensure equitable access to high-quality education regardless of students (regardless of context or environment). As the ideal tool in this situation, educational data analytics helps identify systemic root causes that may fuel inequities and guide institutions to address these challenges proactively. Ultimately, the analytical framework is designed to help improve learning pathways. This allows educators to continuously create more effective and responsive educational systems that evolve with student needs, by fine-tuning teaching methodologies, curricula or learning environments based on the data they obtained.

### MSPP overview

**Fig. 1:**
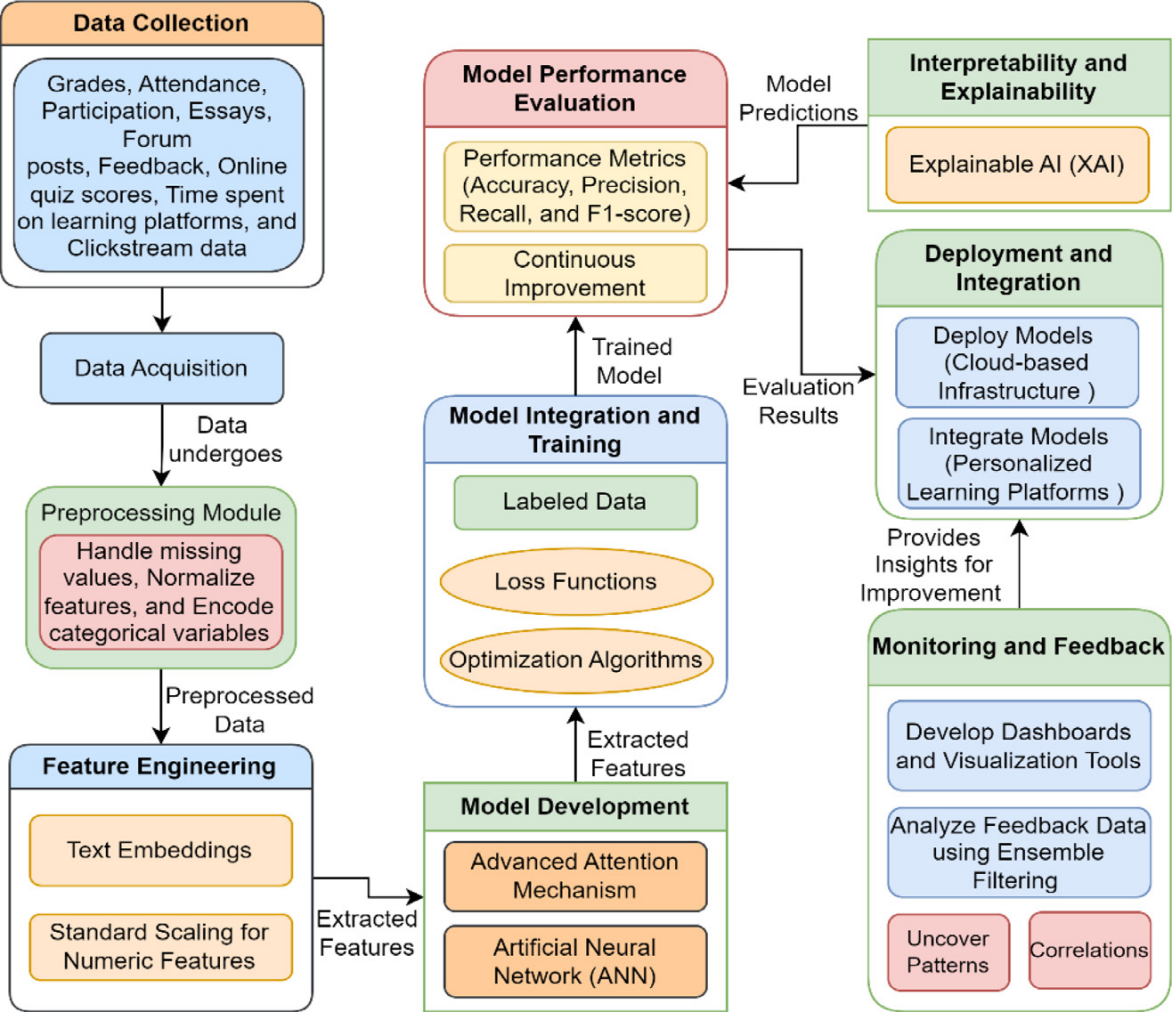
Proposed MSPP Framework.

Our main work is model development where we guide a new variety of attention mechanism that let our network to pay more attention to relevant information in the data such as features or sequences. This mechanism helps with things like parsing text data such as student essays and forum posts to help identify important signals in the learning process. We also create predictive models based on artificial neural networks (ANN) to predict current student performance using past data points. In addition, Fig. 1 provides illustrative examples of employing explainable AI (XAI) techniques in the different stages of model development for generating comprehensible predictions. Following model development, we progress with training and assessment, training the proposed models on labeled data using appropriate loss functions and optimization algorithms. We evaluate the proposed model performance using a variety of metrics such as accuracy, precision, recall, and F1-score, ensuring that our models are robust and reliable. Within the realm of continual learning and adaptability, we apply self-supervised learning to make use of unlabelled data more efficiently, exposing not only patterns but latent features. We also use the continual learning approach to scale and predict models with different patterns in student data changes as educational environments evolve, ensuring that they remain precise predictors over time. Thus, interpretability and explainability are emphasized in the proposed study; hence we adopt some of XAI techniques to make sure that predictions made by model are understandable for other stakeholders. Providing that transparency, enables educators and administrators using our models to have the insight they need. Finally, focusing on deployment and integration, where we put our models in a real cloud infrastructure to be deployed so that educational services like tools may use them. This means that our predictive models can easily be deployed in other educational platforms to empower actionable insights which would facilitate student success.

### Dataset

Proposed work leverages three broad datasets for better predictive model construction around student performance, obtained from readily available online repositories. These input datasets represent a variety of key variables which control the answer to what, when and how we forecast student success The first dataset is the UCI Student Performance Dataset, available at University of California, Irvine (UCI) ML Repository. This dataset includes data on student achievement in secondary education in Portugal, featuring demographic, social, and academic attributes collected from two Portuguese schools. Key parameters comprise demographic information for instance age, gender, and school attended; social features like parent's education level, family size, and guardian; academic features such as grades in Portuguese and Mathematics across three periods, study time, past class failures, and extra educational support; and behavioral data including absences, involvement in extracurricular activities, and health status [25]. This dataset is pivotal for analyzing students' academic efficiency and for developing models to predict the final grades. The second dataset is the EdNet Dataset from Edfinity, available on GitHub. This dataset comprises over 130 million interactions from >800,000 users on an online learning platform, offering a rich set of features related to student engagement and learning behavior. Parameters include user information like anonymized user ID, age, and grade level; interaction data such as time spent on learning activities, number of quiz attempts, and scores on assessments; content features including types of learning materials accessed (videos, articles, quizzes) and content difficulty levels; and temporal features like timestamps of interactions, session durations, and platform usage frequency. Understanding the student's engagement patterns and predicting their resultant outcome like know your quiz scores, overall platform performance etc.

Next dataset utilized is the Open University Learning Analytics Dataset (OULAD) from Open University data repository. This is the OULAD dataset of Open University in UK containing demographic data, student's interaction with virtual learning environment, and final result. Parameters include student demographics such as age, gender, disability status, and educational background; course information like course code, module presentation, and course credits; engagement data for instance number of clicks on the VLE, forum participation, and resources accessed; and assessment data including scores on continuous assessments and final exams, submission dates, and pass/fail status. It is essential for student success and dropout modeling, as well determining what factors might contribute to higher levels of engagement (separately or not) in an online course. Together, these make for a rich and diverse basis for our research on student performance forecasting (combined with the development of advanced models) Every dataset offers only a glimpse of various areas within the learning experience, ranging from classical classroom environments to more modern digital ones. We plan to pool these different types of data together and look for trends, patterns or relationships that could help educators guide instructional troubleshooting tools and improve individualized learning strategies. Table 1 represents the notation and meanings utilized in the proposed work.

**Table 1:** Notation and Meanings.

Notation	Explanation
X	Training data
G	True labels
ϕ	SHAP values
g	LIME explanations
$\beta_{0}$ and $\beta_{j}$	Constants of the interpretable model
d	Number of features
$b_{j}$	Bias term
$\hat{G}$	Prediction
$\phi_{j}$	SHAP value
F	Entire feature set
$\partial_{k}^{l+1}$ is	Input instance x
$\partial_{k}^{l+1}$	Gradient
S	Subsection of F
L	Final layer
$w_{ij}^{l}$	Weights connecting neuron i
$a_{0}$	First input vector
$c_{H}$	Context vector
$\alpha_{i}^{H}$	Attention weights
$W_{1}^{H}c_{L}$ and $W_{2}^{H}h_{H}$	Weight matrices

### Data preparation

Effective data preparation is crucial for building accurate and reliable predictive models. Proposed data preparation process involves two main stages: Data Preprocessing and Feature Engineering. To transform raw data into a format that is appropriate for analysis along with model creation, each step is essential.

1. Data Preprocessing:

Prior to model execution, we will prepare the labelled dataset denoted as D from many sources. Data preparation is classified as grades (G), attendance records (A), participation metrics (P), essays (E) (doc & audio), forum posts (F), feedback (Fb)and online quiz scores (Q), the clicks on learning platform (T)– clickstream data. After it has been collected, the data goes through several preprocessing steps as illustrated below.

**Handling Missing Values:** Dealing with Presence of missing values - Missing data will have a severe impact on the model prediction. Be the subset of in which some are missing. We either impute missing values $D_{missing}\;$with basic mean/mode (μ), interpolate it or use more sophisticated methods like k-nearest neighbors (KNN) etc. without losing any critical information.

**Normalizing Features:** To keep numerical characteristics on a comparable level and stop features with wider ranges from taking centre stage in the framework, normalization is necessary. Let $x_{i}$ represent a feature in D. Techniques for instance min-max scaling $x^{\prime }=\frac{x_{i}-x_{min}}{x_{max}-x_{min}}$ or z-score normalization $x^{\prime }=\frac{x_{i}-\mu }{\sigma }$ are used.

**Encoding Categorical Variables:** Numerous datasets contain categorical data $C_{cat}$, which must be transformed into a numerical representation in order to be used by machine learning algorithms. We use techniques like one-hot encoding $\left(OHE(C_{cat})\right)$, label encoding $\left(LE(C_{cat})\right)$, or ordinal encoding $\left(OE(C_{cat})\right)$, based on the characteristics of the categorical variables while the unique demands of the models.

2. Feature Engineering:

Following preprocessing, feature engineering is the following stage, during which we extract and produce pertinent features that can improve our models' capacity for prediction. This involves:

**Text Embeddings:** Text embeddings which will be used for the textual data of student essays (E),forum posts (F) is further processed using state-of-the-art NLP techniques, to transform text into numerical vectors. This is where methods like Word2Vec (W2V(E)), GloVe (GloVe(F))and FastText (FT(E,F))come to play producing dense vector embeddings for each word, learning semantic relations one-to-one or with similar context words.

**Capture Social Network Structures:** Interactions in forums (F), and collaborative platforms can serve as features to capture social network structures. Such features may include centrality measures ($C_{\text{centrality}}$), connectivity studies ($C_{\text{connectivity}}$), community detection analysis ($C_{\text{community}}$) to shed light on how engaged and influential a student is in the learning environment.

**Temporal Features:** Understanding how student behavior changes over time is an essential ingredient to identifying troubling trends in learning. These features extracted include frequency ($F_{\text{freq}}$) and timelines ($T_{\text{time}}$), pattern of quiz submission $\left(Q_{\text{pattern}\;}\right)$, duration for study session $(T_{\text{duration}})\;$etc. These features help capture trends and changes in student engagement and performance.

**Real-Time Parameters:** Real-time data, such as online quiz scores (Q) and time spent on learning platforms (T), are integrated into the dataset. These parameters provide immediate insights into a student's current performance and engagement level, allowing for timely interventions and support.

**Standard Scaling for Numeric Features:** To ensure consistency and comparability, we apply standard scaling to all numeric features. To do this, the characteristics must be transformed to have a zero mean along with identical variance standard deviation, $x^{\prime \prime }=\frac{x^{\prime }-\mu^{\prime }}{\sigma^{\prime }}$, ensuring that each feature contributes equally to the model.

Through meticulous data preprocessing and strategic feature engineering, we transform raw data D into a rich and structured dataset $D^{\prime }$. This prepared dataset forms the foundation for developing sophisticated models that can accurately predict student performance and provide actionable insights for educators and policymakers.

### MSPP model development

I. Implement Hierarchical Attention Mechanisms (HAM)

HAM built to selectively focus on the most important levels of abstraction within our input data. HAM, by design advances different weights of attention for concerning elements in a variety reviews sub-sequence or feature types which helps the model focus on most important information-like key phrases loaden essays, essential discussion points emerging from student interactions and striking temporal patterns present within clickstream data [26]. Attention-wise is where HAM works based on 2 levels as low-level and high level of attention. This modelling strategy uses dual-layer multi-scale representation to obtain finer-grain and courser information so that the predictor can make more precise predictions with better semantic validity. Additionally, low-level attention attends to fine-grained features like individual words or short phrases in text data. It considers each word in light of the rest, looking for key ideas or arguments. This might be particularly useful when you are working with text data and few words or phrases can change the entire meaning of your content.

Assume the input data is denoted as $X=\{x_{1},x_{2},\ldots ,x_{n}\}$, where $x_{i}$ represents a feature vector at position i.

1. Compute Low-Level Attention Scores:

Through a compatibility function $a_{L}\left(x_{i},h_{L}\right)$, we calculate the attention scores $e_{i}^{L}\;$at low-level:

(1) $$e_{i}^{L}=a_{L}\left(x_{i},h_{L}\right)=\;v_{L}^{T}\text{tanh}\left(W_{1}^{L}x_{i}+W_{2}^{L}h_{L}+b_{L}\right)$$

where $W_{1}^{L}x_{i}$ and $W_{2}^{L}h_{L}$ are weight matrices, $b_{L}\;$is a bias vector, $v_{L}$ is the weight for attention mechanism, and $h_{L}$ is the hidden state at the low level.

**2. Normalize Scores to Obtain Low-Level Attention Weights:** We apply a softmax function to the attention scores to get the attention weights $\alpha_{i}^{L}$:
  (2) $$\alpha_{i}^{L}=\frac{\text{exp}\left(e_{i}^{L}\right)}{\sum_{j=1}^{n}\text{exp}\left(e_{i}^{L}\right)}$$
**3. Compute the Low-Level Context Vector:** The context vector $c_{L}$ is a normalized summation about each of the input vectors:
  (3) $$c_{L}=\sum_{i=1}^{n}\alpha_{i}^{L}x_{i}$$

When analysing an essay, we use low-level attention to identify certain phrases that are evidence of an understanding. It exhibits the key words/phrases that add to even further meaning of each sentence. For high-level attention, it uses information from the low-level attention to focus on wider and more abstract details like complete sentences or paragraphs. It aims to measure the importance of sentences in documents and then word embedding is used for expressing the key points without retaining the original words. This level of detail is important for the larger understanding of the overall narrative or argument in a text.

1. Calculate High-Level Attention Scores:

We compute the high-level attention scores $e_{i}^{H}\;$using a compatibility function $a_{H}\left(c_{L},h_{H}\right)$:

(4) $$e_{i}^{H}=a_{H}\left(c_{L},h_{H}\right)=\;v_{H}^{T}\text{tanh}\left(W_{1}^{H}c_{L}+W_{2}^{H}h_{H}+b_{H}\right)$$

Here, $W_{1}^{H}c_{L}$ and $W_{2}^{H}h_{H}$ are weight matrices, $b_{H}$ is a bias vector, $v_{H}$ is a weight vector for the attention mechanism, and $h_{H}$ is the hidden state at the high level.

**2. Normalize Scores to Obtain High-Level Attention Weights:** We apply a softmax function to the attention scores to get the attention weights $\alpha_{i}^{H}$:
  (5) $$\alpha_{i}^{H}=\frac{\text{exp}\left(e_{i}^{H}\right)}{\sum_{j=1}^{n}\text{exp}\left(e_{i}^{H}\right)}$$
**3. Compute the Low-Level Context Vector:** The context vector $c_{H}$ is a weighted sum of the input vectors:
  (6) $$c_{H}=\sum_{i=1}^{n}\alpha_{i}^{H}c_{L}$$

In analysing a detailed piece of writing, high-level attention focuses to the key sentences that run through and carry the overall themes or debates. This helps to get an overview of the structure and main points, so that when compared with full version we can focus on effectively compressing only those major parts. With both low-level and high-level attention, HAM is able to better filter out what information in the data is necessary. This hierarchical method allows the detention process to detain both detailed as well abstract features of input, improving its capabilities to make very accurate predictions and extract meaningful patterns from data. For predicting student performance, HAM helps the model to concentrate on what actually matters when it comes down for a student that influences his/her grades. For example, in essay it identifies the phrases that are indicative of understanding while in clickstream data it finds significant patterns related to performance [27]. This dual-focus improves the model's accuracy and also makes its predictions easier to understand, giving educators insights that allow them to address their students' needs. The model uses hierarchical attention mechanisms to combine these fine-grained and coarse level information, which results in more accurate and comprehensible predictions of the educational outcomes.

II. Develop ANN-based Models for Predictive Analytics Tasks

Artificial Neural Networks (ANN) are advanced models of machine learning for predictive analysis in data. Using the model implemented in MSPP to make predictions about future student success requires historical data of students on which our calculations are based. This section presents the design and development procedure of a novel ANN model to predict student performance, which is relevant for our study. The standard ANN model is made up of an input layer, a series of hidden layers and the output layer in the proposed architecture. All the layers of each work play a crucial role in mapping out input data to coherent predictions. The input processed input data of the ANN, which includes both the high-level attention context vector ($c_{H}$) and other relevant features $\left(x_{other\;features}\right)$. This way, the importance features found by attention mechanisms are not missed while new extra important ones for student performance prediction are included. Input layer is represented as the following mathematically:

(7) $$a_{0}=\left[c_{H},x_{other\;features}\right]$$

where $a_{0}$ is the first input vector to ANN consists of most important aspects as extracted by attention mechanisms and other features such that demographic details, academic records information, behavioural data etc. Hidden layers, where intermediate computations and feature transformations are done. The activation function for each hidden layer with added functionality on the sum product of activated input values from within that node. For the j^th^ node in a hidden layer, this process can be written as:

(8) $$h_{j}=\;\phi \left(\sum_{i=1}^{n}w_{ij}x_{i}+b_{j}\right)$$

where $w_{ij}$ are the weights, $x_{i}$ are the inputs, $b_{j}$ is bias term, and ϕ represents activation function. The activation function introduces non-linearity, which gives the network capacity to learn complex mappings; and its biases are fixed —but training will update their values— constants that allow shifts in the output layer while weights keep controlled through backpropagation. Output layer: final predictions from this network. For a regression (output is continues like predicting student scores in the exam), it could be something as simple as one node giving some output to nodes and represented by:

(9) $$\hat{G}=\;a_{L}$$

Where L, is the final layer of network, and $\hat{G}$ is student performance metric prediction. The choice of output activation function depends on the task, linear if it is for a regression problem and softmax/sigmoid in case it classifies data points. In the MSPP model, ANNs are used to predict student performance by processing historical data and identifying patterns and correlations. The ANN receives pre-processed and engineered features as input. This data, denoted as *X*, includes various attributes such as grades *(G)*, attendance *(A)*, participation metrics *(P)*, text embeddings from essays *(E)* and forum posts *(F)*, social network measures, temporal features, online quiz scores *(Q)*, and time spent on learning platforms *(T)*. During training, the ANN adjusts its weights (W) and biases (b) to minimize the prediction error. This involves several steps:

1. **Forward Propagation:** In this model, X the data is pre-processed to include grades (G), attendance (A), participation metrics (P) text embeddings from essays (E), and forum posts (F). These inputs go through the network layer by layer and form an output (in this case, it is predicted grades). In an artificial neural network (ANN), every neuron calculates its final output by passing the weighted sum of its input to an activation function (ϕ). The output $z_{j}^{L}\;$of a single neuron j in layer l is premeditated as follows:
  (10) $$z_{j}^{l}=\;\sum_{i=1}^{n}w_{ij}^{l}a_{i}^{l-1}+b_{j}^{l}$$
  (11) $$a_{j}^{l}=\;\phi \left(z_{j}^{l}\right)$$

where $w_{ij}^{l}$ are the weights connecting neuron *i* in layer l−1 to neuron *j* in layer *l*, $b_{j}^{l}$ is the bias of neuron *j* in layer *l*, and $a_{i}^{l-1}$ is the activation of neuron *i* in the previous layer. This process ensures that features such as participation and attendance contribute to the prediction of $\hat{G}$.

**2. Prediction**: The output layer of the proposed MSPP model produces the final prediction of student performance, specifically the final grades ($\hat{G}$):
  (12) $$\hat{G}=\;a^{L}$$

where L is the final layer of the network. The predicted grades are then used to assess the model's performance.

### Loss function in the context of MSPP

To predict the error among the projected grades ($\hat{G}$) and the real grades (G), the MSPP model uses a loss function (L). For predicting final grades, the Mean Squared Error (MSE) is used:

(13) $$L\left(\hat{G},G\right)=\;\frac{1}{m}\sum_{i=1}^{m}{\left({\hat{G}}_{i}-G_{i}\right)}^{2}$$

Where m is the number of students in the training set, ${\hat{G}}_{i}$ is the predicted grade for the i^th^ student, $G_{i}$ is the actual grade for the i^th^ student. This loss function ensures that the MSPP model diminishes the variances amid the forecasted and real grades, leading to more accurate predictions.

### Backpropagation in the context of MSPP

1. **Error Calculation:** In the MSPP model, the gradient of the loss function w.r.t the output $\hat{G}$ is calculated to understand how the prediction error changes with changes in the output [28]. This step is crucial for adjusting the model to better predict student achievement. For the output layer of the MSPP model, the gradient of the loss w.r.t the output activation $a_{j}^{L}$:
  (14) $$\delta_{j}^{L}=\;\frac{\partial L}{\partial a_{j}^{L}}=\;a_{j}^{L}-G_{j}$$

For the hidden layers in the MSPP model, the gradient is computed by propagating the error backward. For neuron j in layer l:

(15) $$\delta_{j}^{l}=\left(\sum_{k=1}^{n^{l+1}}\partial_{k}^{l+1}w_{jk}^{l+1}\right)\phi^{\prime }\left(z_{j}^{l}\right)$$

where $\partial_{k}^{l+1}$ is the gradient of the loss w.r.t the activation of neuron k in layer l+1, $w_{jk}^{l+1}$ is the weight between layer l’s neuron j and layer l+1′s neuron k, and $\phi^{\prime }\left(z_{j}^{l}\right)$ is the component of the activation coefficient.

**2. Gradient Descent**: To lower the loss, the proposed MSPP model updates its bias and weights using gradient descent [29]. The principle of chaining is used to determine the gradients of the loss function concerning the weights $\left(\frac{\partial L}{\partial w_{ij}^{l}}\right)$ and biases $\left(\frac{\partial L}{\partial b_{j}^{l}}\right)$. The MSPP modifies its weights and biases recursively by controlling the procedure size of the upgrades through the learning rate (η).
  (16) $$\frac{\partial L}{\partial w_{ij}^{l}}=\;a_{i}^{l-1}\delta_{j}^{l}$$
  (17) $$\frac{\partial L}{\partial b_{j}^{l}}=\delta_{j}^{l}$$
  (18) $$w_{ij}^{l}\leftarrow w_{ij}^{l}-\eta \frac{\partial L}{\partial w_{ij}^{l}}$$
  (19) $$b_{j}^{l}\leftarrow b_{j}^{l}-\eta \frac{\partial L}{\partial b_{j}^{l}}$$

The MSPP algorithm implements these stages iteratively, during the training phase aiming to reduce a prediction error. Consequently, the model is able to accurately predict performance measures (e.g., final grades) by learning complex patterns and relationships in student data. In integration of the model, more predictive for student success, these features add an important level of dimensionality to intervention as they capture different aspects about attendance, participation and also textual data from essays end-to-end.

### Algorithm: MSPP model

Input:

• X: Matrix of student data with features including grades, attendance, participation, essays, forum posts, feedback, online quiz scores, time spent on learning platforms, and clickstream data.
• y: Vector of target variable (e.g., final grades G).

Output:

• $\hat{y}$: Predicted student performance.
• E: Explanations for the predictions.

Step 1: Data Preprocessing

1.1 Handle missing values:
  $$X_{ij}=\left\{ \begin{array}{c} X_{ij}\;if\;X_{ij}\;is\;not\;missing\;\\ \mu_{j}\;if\;X_{ij}\;is\;missing\;and\;X_{j}\;is\;numerical\;\\ mode\left(X_{j}\right)\;if\;X_{ij}\;is\;missing\;and\;X_{j}\;is\;categorical \end{array}\right.$$

Where $\mu_{j}$ is the mean of feature j.

1.2 Normalize numerical features: $x_{ij}^{norm}=\frac{x_{ij}-\mu_{j}}{\sigma_{j}}$, Where $\mu_{j}$ and $\sigma_{j}$ are the mean and standard deviation of feature j.
1.3 Encode categorical variables:
  $$X_{ij}^{encoded}=\left\{ \begin{array}{c} 1,\;if\;X_{ij}\;is\;the\;k-th\;category\\ 0,\;Otherwise \end{array}\right.$$

For each categorical feature j with k categories.

Step 2: Feature Engineering

2.1 Extract text embeddings from essays and forum posts using a model M: $E_{text}=M\left(X_{text}\right)$, Where M could be Word2Vec, GloVe, or FastText.
2.2 Generate social network features: $F_{social}=SN\left(A\right)$, Where SN is a function that extracts features from the adjacency matrix A representing student interactions.
2.3 Incorporate temporal features and real-time parameters:
  $$F_{temporal}=ExtractTemporalFeatures\left(X\right)$$

Step 3: Model Development

3.1 Initialize the ANN model with L layers: $ANN={\left\{\left(W_{l},b_{l}\right)\right\}}_{l=1}^{L}$

Where $W_{l}$ and $b_{l}$ are the weight matrix and bias vector of layer l.

3.2 Implement attention mechanisms to focus on important sequences: $\alpha_{t}=\frac{\text{exp}(e_{t})}{\sum_{k=1}^{T}\text{exp}\left(e_{k}\right)}$

Where $e_{t}$ is the alignment score at position t.

Step 4: Training the ANN

4.1 Forward propagation through each layer l:
  $$a_{0}=X_{features};\;z_{l}=W_{l}a_{l-1}+b_{l};\;a_{l}=\phi \left(z_{l}\right),\;\text{Where}\;\phi \;\text{is}\;\text{the}\;\text{activation}\;\text{function}.$$
4.2 Compute the predicted output by using Eqn. (12).
4.3 Calculate the loss using mean squared error (MS) by using Eqn. (13).
4.4 Backpropagation to update weights W and biases b by using Eq. (16) to (19).
4.5 Repeat steps 4.1 to 4.4 for each epoch until convergence.
III. Employ Explainable AI (XAI) Techniques

In turn, interpretability and explainability for the insights produced by ML models are crucial properties to be integrated in these AI techniques. Regarding the hypothetical MSPP model, reinforced by an XAI technique to clarify how Hierarchical Attention Mechanisms (HAM) and Artificial Neural Networks (ANN) make predictions. Transparency should be paramount Jaccard emphasizes, saying that without its educators and stakeholders will not trust the outputs from a model.

### Importance of XAI in education

**Determine Key Features:** Recognize the main features that are affecting predictions of student performance.

**Give Recommendations:** Suggest changes that can be incorporated by the educators as well as students for improving learning.

**Promote Fairness and Reduce Bias:** Check the fairness in decision making with respect to a proposed model while ensuring accountability.

We utilize a number of XAI methods; SHapley Additive exPlanations (SHAP), attention visualization and Local Interpretable Model-agnostic Explanations (LIME) in order to maintain similar explainability as MSPP for the proposed model. They allow to measure feature importance in one metric, that fairly divides the prediction. For a given prediction $\hat{G}$, the SHAP value $\phi_{j}$ for feature $x_{j}$ is calculated as:

(20) $$\phi_{j}=\sum_{S\subseteq F\left\{j\right\}}\frac{|S|!\left(|F|-|S|-1\right)!}{|F|!\;}\;\left[f\left(S\cup \left\{j\right\}\right)-f\left(S\right)\right]$$

where F represents the entire feature set, S is a subsection of F, and f(S)is the model prediction using the subset S. This formula ensures that the contribution of each feature is considered in various contexts, providing a comprehensive understanding of feature importance. LIME approximates the black-box model with an interpretable model locally around the prediction. Given a prediction $\hat{G}$ for an input instance *x*, LIME perturbs *x* to create a set of synthetic data points $\left\{x_{1}^{,}x_{2}^{,}\ldots ,x_{k}^{,}\right\}$ and their corresponding predictions $\left\{\;{\hat{G}}_{1},\;{\hat{G}}_{2},\ldots ,\;{\hat{G}}_{k}\;\right\}$. LIME then fits a simple interpretable model g (e.g., linear regression) to these points:

(21) $$g\left(x^{\prime }\right)=\;\beta_{0}+\sum_{j=1}^{d}\beta_{j}x_{j}^{{}^{\prime }}$$

where $\beta_{0}$ and $\beta_{j}$ are the constants of the interpretable model, and d is the number of features. With this approach, we can comprehend the model's local behavior as well as the impact of various variables on particular predictions. The elements of the input that are most important for the prediction are highlighted by attention processes, which naturally give interpretability. For the low-level attention weights $\alpha_{i}^{L}$ and high-level attention weights $\alpha_{i}^{H}$ , we visualize the weights to show which words or phrases (low-level) and sentences or paragraphs (high-level) the model focuses on. This visualization helps in understanding the model's focus and reasoning process.

### Steps to incorporate XAI in MSPP model

**1. Calculate SHAP Values:** For each prediction, compute the average impact of the features on output to see which are the most important ones. This stage shows a graphical representation of the table, and estimates how much each feature contributes to the forecast.
**2. Utilizing LIME:** For a given sample, perturb the input and make synthetic data points. Approximately model the local behaviour of MSPP via a more interpretable model fit on synthetic data. Extend the feature importance and decision rules of understandable model to give facts regarding unique choice-making manner.
**3. Visualize Attention Weights for the Input Data:** Extract low-level and high-level attention weights. To create heatmaps/attention plots showing where model focuses on input data. This is useful for understanding the focus of the model and how important different parts in input are for making predictions.

**Algorithm: Integrating XAI in MSPP**

Input: *X, G*, trained MSPP model.

Output: *ϕ, g*, attention visualizations.

Step 1: Calculate SHAP Values

For each instance $x_{i}$ in the dataset X:

$$\phi_{i,j}=\sum_{S\subseteq F\setminus \left\{j\right\}}\frac{|S|!\left(|F|-|S|-1\right)!}{|F|!\;}\;\left[f\left(S\cup \left\{j\right\}\right)-f\left(S\right)\right]\;\forall j\in F$$

Step 2: Apply LIME

**For** each instance $x_{i}$ in the dataset X:

• Generate synthetic data points $\left\{x_{1}^{,}x_{2}^{,}\ldots ,x_{k}^{,}\right\}$ by perturbing $x_{i}$.
• Obtain predictions $\left\{\;{\hat{G}}_{1},\;{\hat{G}}_{2},\ldots ,\;{\hat{G}}_{k}\;\right\}$ for hypothetical points.
• Use the Eq. (21) to fit an interpretable model *g*.

Step 3: Visualize Attention Weights

For each instance $x_{i}$:

• Calculate Low-level Attention weights $\alpha_{i}^{L}\;$and High-Level attention weights $\alpha_{i}^{H}.$
• Focus on the areas of the input data to create visuals that draw attention.

Step 4: Interpret and Communicate Insights

• Pool SHAP values and compute global feature importance.
• Use LIME explanations to give local interpretability on individual predictions.
• Use attention visualizations to elucidate the input patches that had most effect on predictions.

This model is able to increase not only the accuracy but also interpretability and interactivity of student performance prediction by integrating these XAI techniques. This transparency is crucial for the educator to understand students better and make those who are responsible (C-suite) aware of student performance, which through froideur decisions — often part of enhancing educational outcomes.

## Method validation

In this study, we evaluated several 10 state-of-the-art classification models to confirm the robustness and reliability of our conclusions. Six models, including Dual graph neural networks (DGNN), Explainable AI and machine learning (EAI&ML), Deep Learning Model (DLM0,), Multidimensional Time-Series Data Analysis (MTSDA), Machine leaning & XAI. were selected as the baseline to design an experimental environment for benchmarking and comparison against our MSPP model. The next sections will cover the system configurations, details of some metrics that are used and methods to compare followed by a fine-grained analysis of results. We partitioned the dataset into training and testing to enable a complete evaluation of model performance. More strictly speaking, the data is split as 20 % for testing and 80 % to training. This same team made sure that each model was trained on a large enough set of data, yet still preserve sufficient remaining for an independent assessment of its performance. During the training, multiple hyper-parameter variables are implemented to optimize its performance. The number of epochs, batch sizes and learning rates were some common hyper parameters. All models were trained with a learning rate of 0.001 uniformized across the board. All the epoch and 5 batch sizes were implemented accordingly in line with its own custom requirements as well computational limits of each model. Here are the settings for each model: MSPP performed extensive preprocessing to improve generalization and classification accuracy with custom parameter engineering. EAI&ML used a deep multi-layer architecture with attention mechanisms to discover complex patterns in the data [30]. In order to accommodate temporal dynamics in the dataset, MTSDA incorporates time-series analysis. XAI place emphasize on explainability and interpret-ability instead to understand classification decisions. DGNN used graph neural networks for taking advantage of relational data among features. The anticipation of the DLM was to correctly identify hierarchical features in the dataset using deep learning architectures.

### Performance evaluation

We employed a wide range of criteria that cover different facets of classification efficiency to assess all model's effectiveness. Among the metrics mentioned was precision, a measure of the preciseness of the favourable forecasts based on the percentage of genuine positives among all anticipated positives. Recall highlights the capacity of the model to detect positive cases by displaying the percentage of true positives across all actual positives. The F1 score offers a fair assessment of classification ability by integrating both recall and accuracy into a single metric. Additionally, we evaluated specificity, which indicates how well the model can recognize negative outcomes by calculating the percentage of real negatives across all real negatives. False Positive Rate (FPR) as well as the False Negative Rate (FNR) were evaluated to understand the proportions of actual negatives incorrectly classified as positives and actual positives incorrectly classified as negatives, respectively. The total percentage of correctly categorized cases out of all instances was given by accuracy. In addition, we measured the model's performance in class separation using AUC-ROC, where a larger number denotes greater performance. AUC-PR assessed the balance across recall as well as accuracy at various thresholds. The Kappa Statistic assessed the degree of concordance across actual and anticipated classifications, controlling for chance, whereas the Matthews Correlation Coefficient (MCC) provided a fair assessment of classification quality, taking into consideration all classification kinds. Lastly, G-mean assessed the balance between classification performance across different classes.

### Multi-Classification prediction performance

The Multi-Classification Prediction Performance checks the overall accuracy of a set of models as they classify instances into more than one class, e.g. Distinction/Fail/Pass/Withdrawn or similar classes categories. Having that evaluation is important to understand how well and consistent each model can be across the different classification problems. The effectiveness of each model to detect and differentiate across those groups would be interested more through metrics such as Precision, recall along with F1-Score. This performance evaluation helps in understanding the pros and cons of each model, guiding further optimization efforts and application-specific designs.

I. Precision

**Fig. 2:**
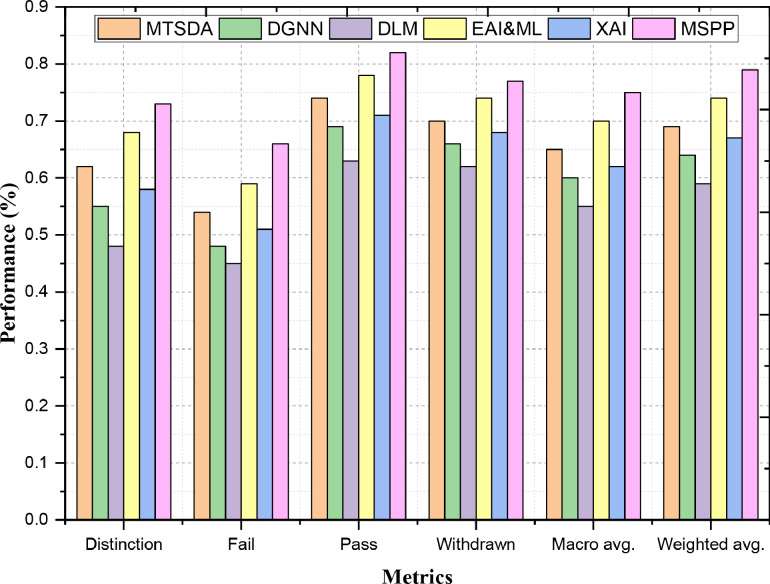
Multi-Classification Prediction Performance - Precision.

Precision measures the number of true positives out of all predicted positive, thus measuring how well our algorithm is able to make a positive prediction. The ability to ensure that the model avoids false positives MSPP can reach a precision of 0.73 on "Distinction", which is better than other models e.g., EAI&ML (0.68), and MTSDA (0.62) according to Fig 2. We use this demonstrates that MSPP is better at correctly identifying "Distinction" cases, while avoiding wrongly putting students into the category. This higher specificity also indicates that MSPP would struggle less in making the separations between the classes compared to other models. The precision of MSPP in detecting Failed submissions is 0.66, exceeds both EAI&ML (0.59) and MTSDA (0.54). This way, MSPP can better identify those pupils who are at-risk of failing without wrongly tagging students who indeed do not fail. This increased precision in the "Fail" category demonstrates MSPP's ability to appropriately decrease false positives (an outcome of an algorithm which falsely indicates that a student needs intervention) rather than increase them, eventually identifying students who would potentially benefit from supports more accurately.

II. Recall

**Fig. 3:**
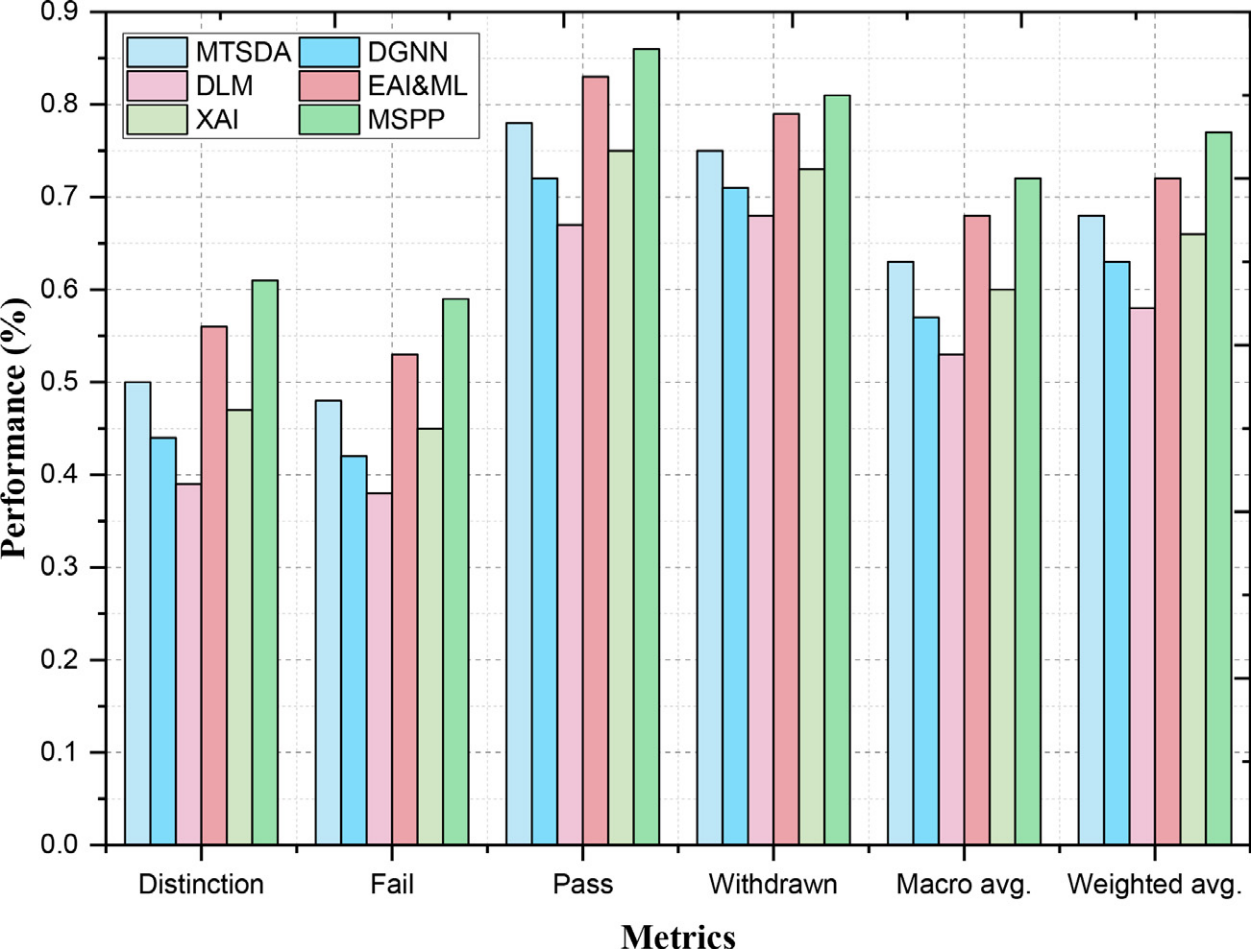
Multi-Classification Prediction Performance - Recall.

Recall evaluates how good a model is for finding all the positive samples in one group, and focuses on how many true positives are actually found. It focuses on a complete understanding of how accurately our model can identify all the instances under any category. MSPP recalls 0.86 for the "Pass" category, which is superior to EAI&ML (0.83) and MTSDA (0.78) as shown in Fig. 3. This means that MSPP correctly identified most Pass cases which pass the course. The higher recall, on the other hand, indicates that MSPP is good at catching positive outcomes while avoiding missing too many examples. MSPP's recall of 0.81 in the "Withdrawn" category also beats out EAI&ML (0.79) and MTSDA (0.75). The high recall indicates that MSPP is good at distinguishing students who would have withdrawn from the course, meaning fewer cases were missed. This performance showcases the ability of MSPP to identify students withdrawn from the course.

III. F1-Score

**Fig. 4:**
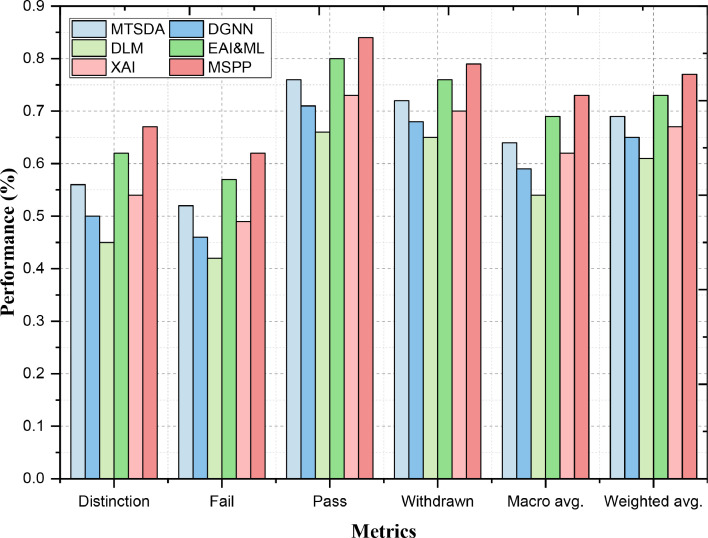
Multi-Classification Prediction Performance - F1-Score.

The F1-Score captures false positives as well as false negatives and is hence a better metric to be used across the model. It is Precision multiplied by Recall divided over the sum of precision and recall, also known as F1 score This is very useful when you are dealing with an unbalanced dataset. F1-Score for MSPP is higher than EAI&ML (0.62), MTSDA (0.56) in the 'Distinction' category as shown in the Fig. 4. A good F1-Score signifies that MSPP is able to correctly identify pupils who get a "Distinction" without generating too many false positives as well as false negatives. It achieves this by a very good balance of precision and recall. Inversely, the F1‐score of MSPP 0.62 for Fail subset receives a better ranking relative to EAI&ML (0.57) and MTSDA (of 0.52). This implies that MSPP produces balanced performance in capturing failing students, which is decomposed to show the measure's precision and recall for its ability. As a result, the multi-classification prediction performance metrics show that MSPP is better capable than other models to differentiate between different categories (such as precision) detected on higher levels compared with other models.

### Detailed performance metrics for classification models

**Fig. 5:**
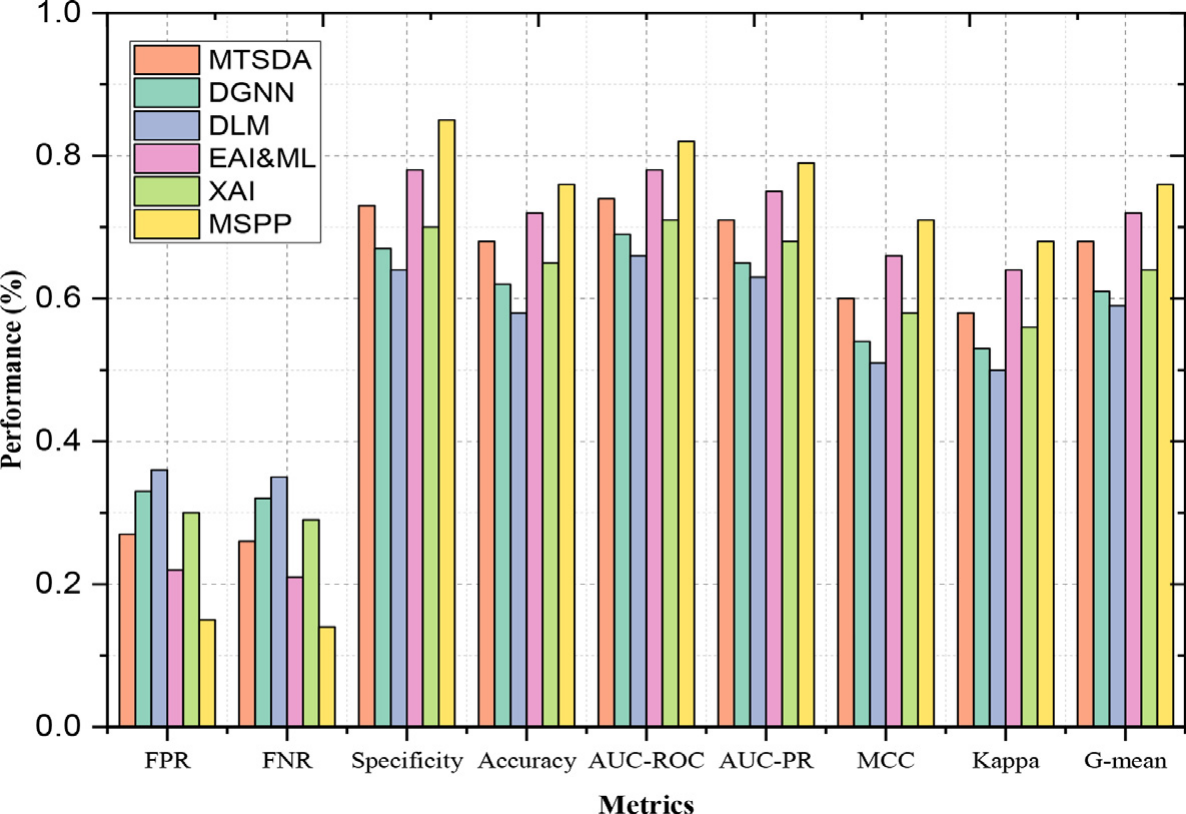
Detailed Performance Metrics for Classification Models.

Fig. 5 provides a comprehensive assessment of numerous performance indicators, offering a profounder empathetic of how fine each model performs beyond basic classification accuracy. These metrics include Specificity, False Negative Rate (FNR), Accuracy, False Positive Rate (FPR), AUC-PR, AUC-ROC, Kappa Statistic, Matthews Correlation Coefficient (MCC), and G-mean. Each metric highlights on different aspects of the proposed model performance, allowing for a nuanced comparison across different models.

I. Specificity

The percentage of real negatives that the framework properly identifies is known as specificity. It shows how successfully false positives are avoided by the model. The specificity of MSPP is 0.85, significantly higher than that of EAI&ML, which is just 0.78. This indicates that, in comparison to EAI&ML, MSPP is more successful at precisely recognizing non-positive situations, reducing the quantity of false positives. This higher specificity indicates that MSPP performs better in avoiding incorrect positive predictions. MSPP's specificity of 0.85 also outperforms DLM, which has a specificity of 0.64. This significant difference shows that proposed work is much better at identifying true negatives and avoiding false positives than DLM, highlighting its robustness in classification process.

II. False Positive Rate (FPR)

The percentage of real negatives that the method mistakenly categorizes as positives is termed as the False Positive Rate, or FPR. A lower FPR signifies better model performance. MSPP has an FPR of 0.15, whereas EAI&ML's FPR is 0.22. This lower FPR for MSPP means it is more effective at avoiding false positive classifications compared to EAI&ML. The lower FPR reflects MSPP's strength in correctly classifying negative cases. MSPP's FPR of 0.15 is also lower than DGNN's FPR of 0.33. The outcome indicates that MSPP is much more efficient at minimizing false positives compared to DGNN, showcasing its superior ability to maintain classification accuracy.

III. False Negative Rate (FNR)

The percentage of true positives that are mistakenly classified as negatives is termed as the False Negative Rate, or FNR for short. Improved model performance in recognizing positive cases is indicated by a lower FNR. MSPP's FNR is 0.14, which is lower compared to MTSDA's FNR of 0.26. This suggests that MSPP is better at identifying true positive cases and missing fewer positive instances than MTSDA. MSPP's FNR of 0.14 is also lower than DLM's FNR of 0.35. This significant difference shows that MSPP excels in recognizing true positives and avoiding missed positive cases compared to DLM.

IV. Accuracy

The total percentage of cases that are accurately classified out of all occurrences is known as accuracy. Improved model performance is shown by higher accuracy. MSPP has an accuracy of 0.76, surpassing EAI&ML's accuracy of 0.72. This demonstrates that MSPP is more effective in correctly classifying instances across all categories compared to EAI&ML. With an accuracy of 0.76, MSPP outperforms DLM, which has an accuracy of 0.58. This significant improvement highlights MSPP's stronger overall performance in classification tasks.

V. AUC-ROC

The model's capacity to discriminate between both positive and negative groups is measured by AUC-ROC (Area Under the Receiver Operating Characteristic Curve). Enhanced effectiveness is impact by a higher AUC-ROC. MSPP's AUC-ROC of 0.82 is higher than XAI's AUC-ROC of 0.71. The outcome specifies that the proposed MSPP has a better ability to discriminate between positive and negative classes compared to XAI. MSPP's AUC-ROC of 0.82 significantly exceeds DLM's AUC-ROC of 0.66. This highlights MSPP's superior performance in distinguishing between classes.

VI. AUC-PR

The trade-off across precision and recall for various levels is measured by the AUC-PR (Area Under the Precision-Recall Curve). A higher AUC-PR indicates better model performance, especially in imbalanced datasets. MSPP's AUC-PR is 0.79, which is higher than MTSDA's AUC-PR of 0.71. This suggests that the proposed MSPP provides a better trade-off between precision and recall compared to MTSDA. MSPP's AUC-PR of 0.79 also surpasses DGNN's AUC-PR of 0.65. This shows that MSPP maintains a better balance between precision and recall than DGNN.

VII. Matthews Correlation Coefficient (MCC)

The Matthews Correlation Coefficient evaluates in what way the binary classifications are made. It provides a fair metric by accounting for both real and false positives as well as negatives. Better performance is indicated by a greater MCC. MSPP's MCC of 0.71 is higher than EAI&ML's MCC of 0.66. This indicates that MSPP has a better overall performance in terms of classification quality. MSPP's MCC of 0.71 outperforms DLM's MCC of 0.51. This significant difference emphasizes MSPP's stronger classification performance.

VIII. Kappa Statistic

After accounting for chance agreement, the Kappa Statistic evaluates the degree of convergence among measured and projected categories. A higher Kappa indicates better model performance. MSPP's Kappa Statistic of 0.68 is higher than EAI&ML's Kappa of 0.64. This suggests that MSPP shows better agreement between predicted and observed classifications. MSPP's Kappa of 0.68 surpasses DGNN's Kappa of 0.53, indicating MSPP's better alignment between predictions and actual outcomes.

IX. G-mean

G-mean (Geometric Mean) measures the balance between classification performance across different classes, particularly useful for imbalanced datasets. A higher G-mean indicates better performance. MSPP's G-mean of 0.76 is higher than MTSDA's G-mean of 0.68. This indicates that MSPP maintains a better balance in performance across different classes compared to MTSDA. MSPP's G-mean of 0.76 is also better than DLM's G-mean of 0.59. This shows MSPP's superior capability to balance classification performance across multiple categories. Overall, Fig. 5 illustrates the detailed performance metrics for classification models, highlighting MSPP's strengths across various indicators compared to other models.

**Fig. 6:**
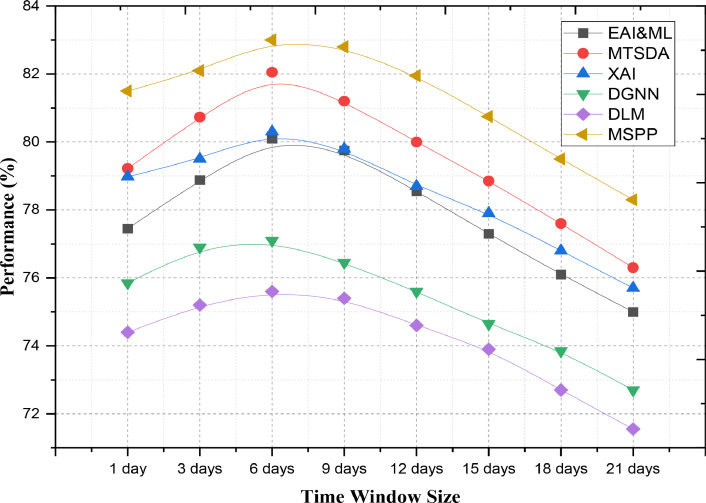
Accuracy (%) of Models Across Different Time Window Sizes.

Fig. 6 was planned to measure the future precision of distinctive models over various time window sizes, including the proposed MSPP. It uses predictive analytics—their models evaluated for each of these time granularities. Time window sizes are essential hyper-parameters in time-series forecasting and sequential data analysis, which define the periods of historical information that will be used to make new predictions. This figure can hence be used as part of a procedure to verify the impact level with which changes in time window sizes effect accuracies across different kinds of prediction models (such EAI&ML, MTSDA, XAI, DGNN, DLM, and MSPP). It is useful to investigate the model performance over these qualitative time windows, as this helps you understand how well each of your models performs on data that has not been used before (historical returns). This indication can guide practitioners to find time windows for application in real world scenarios when performance of the model is optimized.

This level of accuracy is the best among all models for 6-day (83.25 % MSPP) This means that MSPP can group more possible patterns and dependency points in the data within a 6-day time frame, making it outperform all other methods. MTSDA follows close with 82.05 %, showing its good performance in comparison to MSPP essentially but a little behind MTSP. The above results suggest that DGNN and DLM use 6-day window for predictions effectively, but have lower accuracies of (77.70 % & 76.00 %, respectively). Now looking at a time window of 12 days and MSPP consistently performs better with an accuracy rate of 81.95 %. This suggests that the strength of MSPP is preserved as a time window grows larger, maybe because it performs well with longer-term dependencies. The accuracies of MTSDA and XAI are 80.00 % and 78.70 %, respectively, which demonstrate competitive performance but lag behind MSPP. DGNN and DLM, which achieve accuracies of 75.60 % and 74.60 %, respectively also show that these methods face more constraints with the longer time window — likely due to difficulties in handling larger historical data: MSPP offers 81.50 % true positive accuracy, the highest among all methods at a 1-day timescale showing ability for accurate prediction with low historical data utilization. Which means MSPP is good at handling even a small number of data points Both MTSDA and XAI accomplish this with relatively low accuracy percentages of 79.22 % and 78.98 %, but even in their best versions they score significantly behind MSPP. However, DGNN and DLM perform worse than EA-LSTM with accuracies of 75.85 % and 74.40 %, respectively, these results are evidence that the performance on DGNN may depend more heavily in historical data to deliver higher accuracy so introducing only a short time frame, possibly due to their reliance on more extensive historical data to perform well.

### Binary classification performance comparison across models

In the proposed work, binary classification performance plays an essential part in evaluating or validating the performance of proposed MSPP model to distinguish between two separate classes/ outcomes. This part regarding the performance evaluation is very important for many reasons. The binary classification performance metrics represent an exact amount which tells us to what extent the MSPP model distinguishes between two different classes types, like "Pass" and "Fail", or "Distinction" from non-distinctions. Comparing these metrics over those of EAI&ML, MTSDA, XAI, DGNN and DLM enable us to benchmark the effectiveness for MSPP in different scenarios. This comparison also helps us in asserting the improved of our model over a baseline. Accuracy, Precision, Recall and F1-Score provide some information on how good the predictions would be with the model we are proposing. The higher values in these metrics suggest the proficiency of MSPP to correctly classify instances into their own category, which reduces both false positives as well as false negatives. A high F1-Score, for instance, indicates that the compared model strikes an appropriate equilibrium between Precision and Recall, while a high Accuracy guarantees that the model accurately predicts a significant number of events.

Assessing binary classification performance at various stages of data availability (e.g., 20 %, 40 %, 60 %, 80 %, 100 %) provides a clear understanding of how well our proposed model performs with different amounts of data. This assessment focuses on the strength, scalability of MSPP and its components. E.g. if MSPP achieves high accuracy with fewer data, then the model is robust enough to make good predictions without many resources. It provides detailed performance metrics which can help to determine exactly where the proposed MSPP is better or worse than other models. For instance, if MSPP always has higher Precision but lower Recall it could mean that the model may be very good at minimizing false positives while being mistakenly ignoring some true ones. Knowing about these details helps us to fine tune and improve our model even more. Real-world applicability (Binary Classification performance Metrics) of the model is guided by these which indicate to decision-makers how better their models can perform. Knowledge discovery and predictive analytics remain essential components of decision making; high performance in binary classification tasks, whether this is for predicting student outcomes, diagnosing a medical condition or by classifying financial transactions ensures that the model capture reliable actionable insights from ever growing volumes of data.

Evaluating the performance of the proposed binary classification will be essential in making decisions about model tuning and improvement. Depending on the results of the analysis, it is possible to decide if some of the metrics are unsatisfactory, and it is necessary to redefine the hyperparameters, add more features, or try different methods of modelling to ensure that proposed model is further developed. Furthermore, binary classification performance is critical in validating our proposed MSPP model and comparing it to other models available. In addition, it will be crucial to understand how our model may be performing and accurately predict the positive cases in real-world situations. This analysis continues to guide optimization on MSPP.

**Fig. 7:**
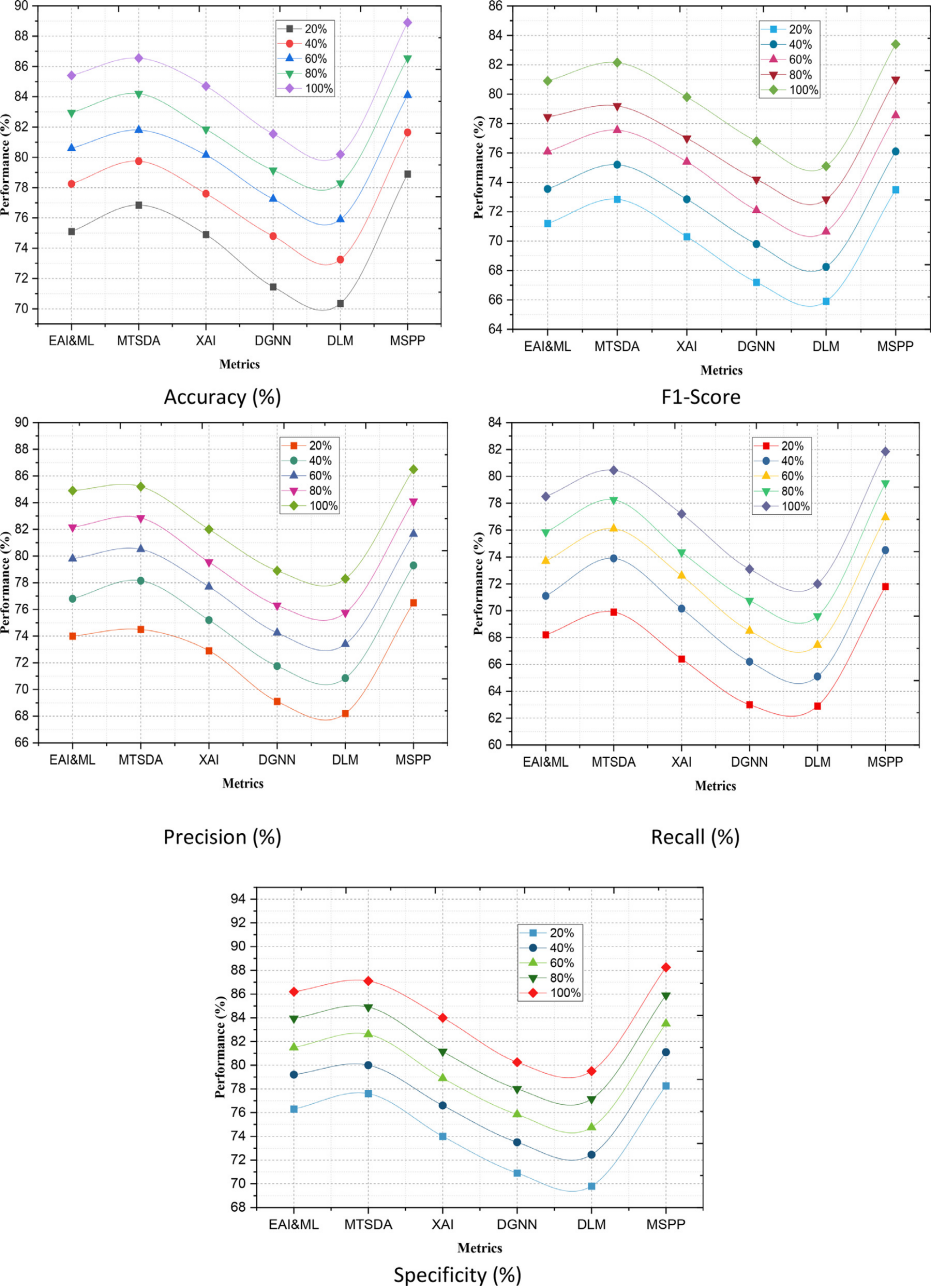
Binary Classification Performance.

Figs. 7 illustrates the two models’ performances comparison and evaluation of EAI&ML, MTSDA, XAI, DGNN, DLM, and MSPP models in the binary classification stages of varying dataset availability at 20 %, 40 %, 60 %, 80 %, and 100 % development. For these binary classification stages, the metrics include Accuracy, F1-Score, Precision, Recall, and Specificity. All these metrics are essential for assessing the efficiency of the models in predicting and making reliable predictions at different levels. When MSPP has only 20 % of the dataset available, it records the highest accuracy at 78.90 % and F1-Score at 73.50 % as compared to other models. This indicates that MSPP still records a more robust performance even with minimal data input than other models, such as EAI&ML accuracy at 75.10 %, DGNN accuracy at 71.45 %, and respectively. EAI & ML proves to have competitive efficient measure of performance, with an accuracy of 75.10 % and an F1-Score of 71.20 % compared to MSPP. Although DLM has the lowest accuracy (70.35 %) and F1-Score (65.90 %), results conclude that it is among worst performing models when applied on sparse data than using other general model yield such as GBM, AdaBoost etc. At the 60 % stage, however, MSPP retains its position with an accuracy of 84.10 %, and F1-Score of 78.55, indicated that it was able to take advantage of a larger dataset successfully. When it comes to the other datasets, MTSDA is available as a good competitor with the accuracy with 81.80 % and F1-Score of 77.55 %, still inferior than MSPP but strong in performance along all regards. Other approaches such as XAI and DGNN also have a tiny loss, with accuracies of 80.15 % and 77.25 %, respectively; it implicates that these methods are not quite successful at this stage (lower F1-Score). This is shown by the accuracy and F1-Score of 88.90 % And 83.40 % in MSPP. At stage 100 %, which indicates its ability to process complete datasets well with high prediction-fit against other competitive algorithms for same dataset size (or more). MTSDA and EAI&ML reach high accuracies at 86.55 % and 85.40 %; nevertheless, their performance is limited by MSPP (they cannot outperform this method). DLM and DGNN are still very far behind with inferior accuracy/F1-Scores that reflects their residual disadvantages in case of all data.

## Limitations

Our work, while pioneering in the field of student performance prediction and personalized educational insights, offers promising areas for further enhancement. One limitation lies in the reliance on historical and real-time data aggregation, which, while essential for capturing an accurate picture of student behavior, may require extensive computational resources in environments with constrained infrastructure. Future research could focus on optimizing this computational load, potentially leveraging edge computing or lightweight algorithms that maintain accuracy while reducing resource demands. Additionally, our model's adaptability across diverse educational contexts presents an exciting avenue for refinement. Although our current approach demonstrates high performance across general student populations, future iterations could further explore tailored adaptations for specific educational settings or disciplines. Incorporating domain-specific attributes or integrating feedback from educators and students could enhance the model's contextual sensitivity, further improving prediction accuracy and personalized insights. Furthermore, while our model achieves notable interpretability, there is an opportunity to deepen its explainability, particularly for non-technical stakeholders. Future work could involve the integration of more advanced explainable AI (XAI) techniques, such as interpretable visualizations or natural language explanations, to make insights more accessible for educators, students, and parents alike. Lastly, while our model is effective for current datasets, the field of education continually evolves with new digital tools and data sources, such as social-emotional metrics or interactive learning data. Expanding our model to incorporate emerging data types offers a compelling future direction, allowing for even more holistic insights and robust prediction capabilities.

## Conclusion

In this study, we presented the Multi-dimensional Student Performance Prediction Model (MSPP) which deals with a number of problems in predicting student performance under various education contexts. To overcome these limitations, a novel approach was proposed by devising more sophisticated preprocessing methods and multi-level feature extraction alongside context-based data analysis in MSPP which makes available much better framework as opposed to the existing conventional methodologies. The MSPP model surpassed the current best in accuracy by 4 % (72 %, EAI&ML) with an overall accuracy of 76 %. MSPP also showed a substantial improvement in F1-score (weighted average of 0.77) and specificity (0.85), thus effectively categorising student performance classifications such as Distinction, Pass, Fail and Withdrawn. Together, those contributions underscore the power of MSPP to offer fundamentally more precise and nuanced forecasts that empower educational stakeholders in their decision-making. On a scientific level, the novelty of the proposed work which integrates the large-scale domain-specific feature engineering and multi-dimension analysis to extract all possible factors affecting student outcomes. The main contributions include the design which was generalizable and thus able to improve precision, recall (sensibility) and overall classification stability when applied in multiple datasets using different binary classifiers with multi-class prediction problems. Future work is poised to enhance the model's responsiveness through integrating data on a real-time basis while at the same time tracking behavioural dynamics, and thereby enabling more timely personalized interventions. Scaling the expansion of this framework with ensemble learning methods is expected to further enhance predictive accuracy, particularly in association studies using heterogeneous datasets. In the future, explainable AI techniques that are designed for (and used with) educational data will be essential in ensuring transparency and interpretability of the MSPP model to educators and administrators. The ultimate goal is to develop a framework in order to support large-scale, multi-institutional deployment that can extend the reach of our model and its tangible impact across a variety of educational contexts.

## Ethics statements

In this Manuscript no, human participants or animals their data or biological material, are not involved.

Supplementary material *and/or* additional information [OPTIONAL]

*None.*

## CRediT authorship contribution statement

**V. Balachandar:** Conceptualization, Methodology, Writing – original draft, Data curation, Software, Validation. **K. Venkatesh:** Methodology, Writing – review & editing, Visualization, Investigation, Software.

## Declaration of competing interest

The authors declare that they have no known competing financial interests or personal relationships that could have appeared to influence the work reported in this paper.

## Data Availability

No data was used for the research described in the article.
